# Rapid Brain Death following Cardiac Arrest without Intracranial Pressure Rise and Cerebral Circulation Arrest

**DOI:** 10.1155/2018/2709174

**Published:** 2018-07-19

**Authors:** Maxime Nguyen, Thomas Bièvre, Abdelouaid Nadji, Bélaïd Bouhemad

**Affiliations:** ^1^Department of Anesthesiology and Intensive Care, C.H.U. Dijon, Dijon Cedex, France; ^2^Université Bourgogne Franche-Comté, LNC UMR866, 21000 Dijon, France

## Abstract

We describe here an unusual case of brain death following cardiac arrest. Brain electric activity had totally ceased, allowing the confirmation of brain death, despite normal cerebral blood flow (assessed by both transcranial doppler and tomodensitometry) and no evidence of intracranial hypertension. In our case, a residual electric activity was assessed at admission and lesions worsened on imaging during ICU stay, suggesting that part of the neuronal damage occurred after brain reperfusion. All these elements suggest BD rather by cellular toxicity than intracranial pressure elevation.

## 1. Introduction

Brain death is defined by complete and irreversible loss of brain function. The determination of brain death (BD) is critical because BD is the main condition allowing organ retrieval. There is no international consensus over criterion for BD diagnosis and especially on weather ancillary testing should be systematically performed [1]. In France, BD diagnosis is defined by law and both clinical examination and ancillary testing are required before BD declaration [2].

## 2. Case Report

A 61-year-old patient was admitted after cardiac arrest. Low-flow duration was of 20 minutes and no-flow duration was brief. The cause of the cardiac arrest remained uncertain. The patient's hemodynamic status remained stable throughout the ICU stay, only requiring low-dose norepinephrine for less than 24 hours.

**Figure 1:**
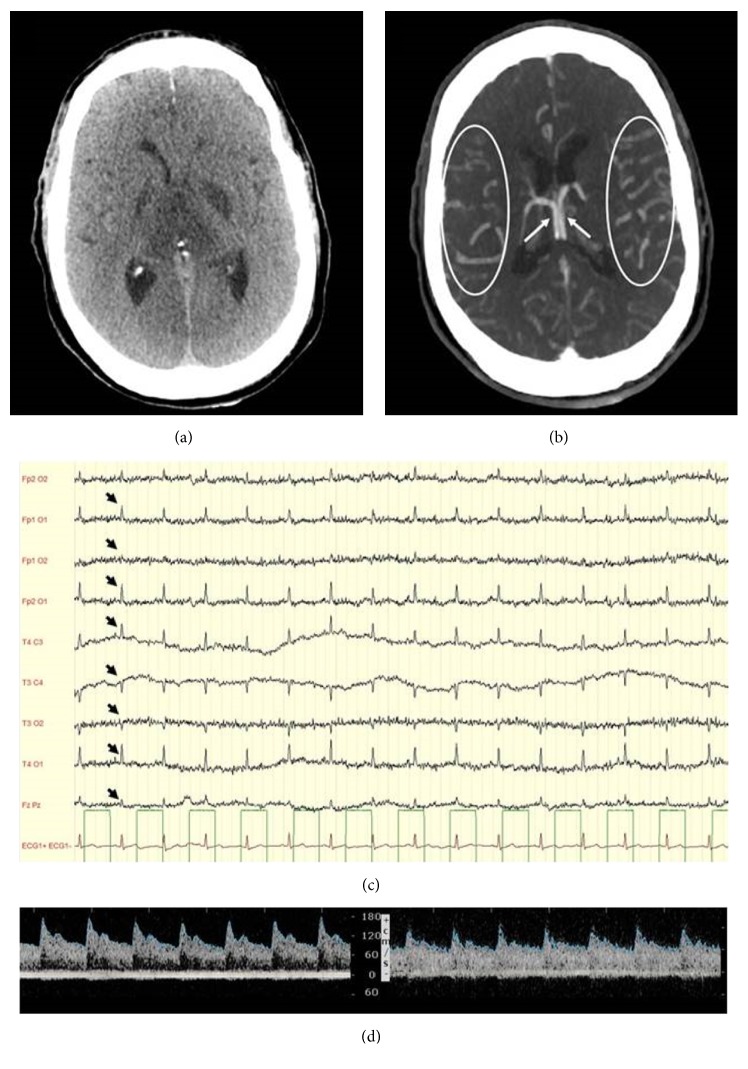
(a) Cerebral CT scan at H76. Brain tissue shows a loss of differentiation between white matter and gray matter. Lateral ventricles are preserved and Sylvian fissures are visible. (b) Cerebral CT angiography. Bilateral opacification of the internal cerebral veins (arrows) and of the cortical (M4) segment of middle cerebral arteries (circled). The four nonopacification criteria used to confirm brain death are absent. (c) Electroencephalogram. Absence of brain electrical activity, including stimulation, on all derivations confirmed on two 30-minute EEGs done 4 hours apart confirmed brain death. Cardiac electric activity is seen on the EEG (black arrow) synchronized with the actual electrocardiogram (red line). (d) Transcranial Doppler of the right and left middle cerebral arteries. The cerebral blood flow has a normal aspect. There are no signs of intracranial hypertension as diastolic velocities are over 40 cm/s and the Pulsatility Index is above 1.4.

At admission (H0), the patient was unresponsive with preserved brainstem reflexes. Unresponsive anisocoria (left myosis) was also noted. The initial CT did not reveal any sign of elevated intracranial pressure (ICP). A first electroencephalogram (EEG) showed no status epilepticus and slow but persistent activity. At H72, brainstem reflexes disappeared. Second EEG was flat. In the meantime, transcranial Doppler (TCD) examination of median cerebral arteries showed preserved cerebral blood flow with normal diastolic velocity. On the 6th Day, sedative drug assay was negative. The apnea test was positive. Third and fourth EEG (Figure 1(c)) confirmed brain death. At that time, the TCD patterns remained unchanged (Figure 1(d)). A CT angiography revealed a loss of white matter/gray matter differentiation and a normal 4-point opacification score (Figures 1(a) and 1(b)). The volume of the lateral ventricles was preserved and the Sylvian fissures were still visible. Brain death was declared 145 hours after admission according to French law [2, 3].

## 3. Discussion

We describe here an unusual case of brain death occurring after cardiac arrest. Brain electric activity assessed by EEGs had totally ceased, allowing the confirmation of BD, despite normal cerebral blood flow (CBF) and no evidence of intracranial hypertension.

EEG was one of the first ancillary tests described for BD diagnosis [4]. Whereas false negative rates for BD due to agonal activity are frequent, false positive rates of EEG have been poorly described and are caused either by surviving subcortical neuron or by confounding conditions [5]. Regarding cerebral blood flow, CTA might show a persistent perfusion despite the absence of perfusion on angiography [6]; however the divergence is mostly explained by residual perfusion in proximal arteries, which are not considered in the actual 4-point opacification score [7]. TCD has been described to have a specificity as high as 99% leaving false positive cases anecdotic [8]. In our case, EEG and TCD pattern were characteristics with normal diastolic velocities and no residual electrical activity. Furthermore, whereas most of previous reports showed impaired CBF, supporting a premature assessment of CBF prior to cerebral circulatory arrest [9–11], in our study, CTA showed preserved perfusion in both proximal and distal arteries and no sign of evolution toward cerebral circulatory arrest was observed.

White matter/gray matter dedifferentiation has been described as a sign of ischemia and is associated with a poor neurological outcome in cardiac arrest. It is probably the consequence of cytotoxic edema in the gray matter and indicates severe diffuse anoxic cortical injury [3, 4]. In our case, this aspect was consistent with extensive cortical lesions and could explain the absence of brain electrical activity. The preserved volume of the lateral ventricles along with visible Sylvian fissures ruled out elevated intracranial pressure and explained the persistence of CBF.

Even though persistent CBF might indicate preservation of cerebral hemisphere, persistence of CBF might be observed in brain dead patient, dissociated from neuronal damage. The most striking example is being observed in patient with craniectomy for whom CBF is restored despite previous cerebral circulatory arrest [9, 12]. Similarly, during cardiac arrest, when CBF drops below a certain threshold, neuronal lesions occur [13]. After return to spontaneous circulation, CBF is restored. However, as the EEG at admission showed residual electric activity, and as the lesions on imaging worsened between admission and BD, one can hypothesize that part of the neuronal damage occurred after brain reperfusion in our ICU.

To the best of our knowledge, our case is the first to combine EEG, TCD, and CT angiogram at the same time, to illustrate complete dissociation between cerebral blood flow and electric activity. All these elements suggest, in our patient, BD after cardiac arrest rather by cellular toxicity than ICP elevation.

Because CBF was preserved, no apnea test and no second EEG were performed at H72 despite the absence of brain stem reflexes and a first flat EEG. This unfamiliar evolution probably delayed BD diagnosis for several days.

## 4. Conclusion

Our report suggests that, following cardiac arrest, brain death might occur despite a normal cerebral blood flow and in absence of ICP elevation. In our patient, part of neuronal damage occurred after brain reperfusion. EEG is the ancillary test allowing brain death confirmation in such settings.
